# Good CoP, bad CoP? Interrogating the immune responses to primate lentiviral vaccines

**DOI:** 10.1186/1742-4690-9-80

**Published:** 2012-10-01

**Authors:** Per Johan Klasse, John P Moore

**Affiliations:** 1 Department of Microbiology and Immunology, Weill Cornell Medical College, Cornel University, 1300 York Avenue, Box 62, New York, NY, 10065-4896, USA

**Keywords:** Correlates of protection (CoP), Neutralizing antibodies, Mucosal immunity, *In vivo* mechanisms, SIV, HIV, Vaccine trials, RV144, Synergy, Innate immunity

## Abstract

Correlates of protection (CoPs) against infection by primate lentiviruses remain undefined. Modest protection against HIV-1 was observed in one human vaccine trial, whereas previous trials and vaccine-challenge experiments in non-human primates have yielded inconsistent but intriguing results. Although high levels of neutralizing antibodies are known to protect macaques from mucosal and intravenous viral challenges, antibody or other adaptive immune responses associated with protection might also be mere markers of innate immunity or susceptibility. Specific strategies for augmenting the design of both human trials and animal experiments could help to identify mechanistic correlates of protection and clarify the influences of confounding factors. Robust protection may, however, require the combined actions of immune responses and other host factors, thereby limiting what inferences can be drawn from statistical associations. Here, we discuss how to analyze immune protection against primate lentiviruses, and how host factors could influence both the elicitation and effectiveness of vaccine-induced responses.

## Review

### Contradictory trends in pre-clinical and clinical research on HIV-1 prevention

A goal of medical research is to find causes: as noted already by Claude Bernard, statistical patterns are an insufficient basis for saving lives [1]. This insight should guide research on prevention of HIV-1 transmission, which remains urgent because every year 2–3 million more people become infected. Of four large-scale human vaccine efficacy trials, only the fourth showed some statistically subtle protection, although each of them has yielded intriguing statistical associations. None has shown dampening of viral replication in cases of infection [2-5]. In contrast, control of viremia is regularly achieved by different vaccine approaches in the simian experimental models of HIV-1 infection [6-10]. There, cytotoxic T-lymphocyte (CTL) responses generally correlate with viremic control, whereas protection from infection is linked to neutralizing antibodies (NAbs) [6,9,11]. Protection by NAbs has also been directly demonstrated through passive immunization of macaques [12-14], but vaccination of humans or animals has not yet elicited broadly active potent neutralization responses [15].

An overview therefore yields a paradoxical picture: the CTL responses that are readily induced in humans have not dampened viral loads while the, at most, modest protection afforded by one vaccine occurred in the absence of strong and broad neutralizing responses. Furthermore, NAb responses, although active against only a subset of sensitive HIV-1 strains (tier 1), were induced more strongly in an earlier human trial in which there was no protection from infection [16]. The outcomes of human trials have sometimes been explored through new studies in non-human primates. Vaccine experiments have also been interpreted in the light of post-trial analyses that have identified immune variables associated with distinct risks [3,4,6,9,16-18]. Here we compare the outcomes of human trials and animal experiments, and we discuss how the design and analysis of both types of vaccine research could be improved.

### Defining causes and correlates of protection

Statisticians and vaccinologists have used the terms *surrogate* and *correlate of protection* in contradictory ways. The terminology was recently unified, although omitting the term *surrogate*[19]. In a vaccine trial or experiment, a correlate of risk (CoR) is the broadest term, covering both reduced and increased risk. It includes immune responses to the vaccine that correlate with a low risk of the endpoint, be that acquisition of infection or an elevated viral load. A correlate of protection (CoP) is a special case of an inverse CoR: a CoP must be responsive to alteration through vaccination, and it must predict the vaccine efficacy based on comparisons of vaccinees and controls. A CoR in the vaccine group can be hypothesized to be a CoP, but a CoR is not a CoP if it merely tracks pathogen exposure or intrinsic susceptibility to infection or disease. Hence a genetic CoR (in the case of HIV-1 infection, for example, homozygozity for the *CCR5Δ32* allele) cannot be a CoP, although it may confer better protection than any vaccine.

There are two kinds of CoPs: mCoPs (mechanistic) constitute the protective immune variable itself, while nCoPs (non-mechanistic) are statistically associated with the mechanistic factor without directly conferring protection. Thus an nCoP can, for example, share a cause with the mCoP, be caused by the mCoP, or contribute as a partial, possibly necessary but not sufficient, cause of the mCoP.

The two kinds of CoP are suggested to be mutually exclusive [19]. This distinction can, however, become intricate. For example, if mucosal IgG NAbs protect against vaginal transmission, and IgG in the vaginal mucosa is largely transudated from plasma, would plasma IgG NAbs then be merely an nCoP? The answer depends on how many links in the causal chain one elects to include in the mechanism of protection. In practice, because plasma and vaginal IgG titers are imperfectly correlated, plasma NAbs might be a poor predictor of protection against vaginal challenge, i.e. not even an nCoP [20]. Furthermore, NAbs are a subset of total Env-reactive Abs. If NAbs constitute the mCoP, and there is a correlation between neutralizing and total Env-specific Abs, then the nCoP would be what remains of the total Env Abs after subtraction of the NAb component. In that example, the inclusive CoP and the mCoP would be more easily determined than the nCoP. In other cases, an nCoP might be more readily detected. For example, CD8^+^ effector memory T-cells (T_EM_) could be analyzed in bronchoalveolar lavage and used to track the mCoP, i.e., the corresponding prevalence of such cells at the less readily accessible rectal site of virus deposition [8]. When the mCoP involves multiple factors, however, each by itself unable to tip the balance against infection, the mutual exclusivity criterion again becomes problematic. Thus, although each factor individually might be a good marker without being mechanistically sufficient, and hence would qualify as an nCoP, it would still be a component of, and therefore overlap with, the mCoP.

### Divergent outcomes of human trials

**Figure 1:**
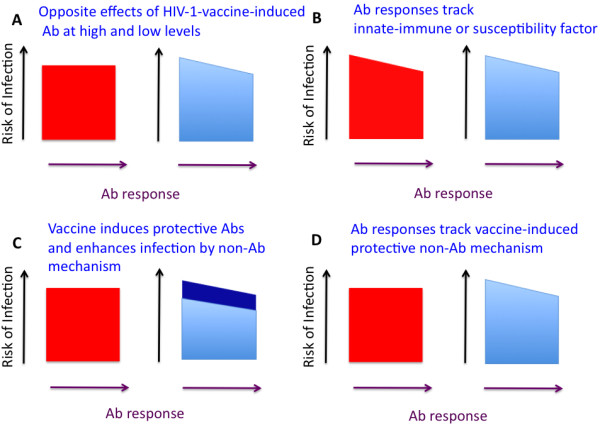
**Dissecting direct from indirecteffects of vaccination bycomparing with irrelevant immunization.** Each square or trapezoid (placebo controls in red, vaccinees in blue) represents the risk of infection on the y-axis. Ab response to the HIV-1 (vaccinees) or irrelevant immunogen (both groups) is represented on the x-axis. The vaccine gives zero net protection: the areas of the squares and trapezoids are identical. **A**. Strong Ab responses to HIV-1 immunogens reduce and weak ones increase risk. The risk of infection does not vary with the response to an irrelevant immunogen for the placebo cases. **B**. The risk varies similarly with Ab responses to the irrelevant immunogen in placebo and vaccine groups. The associated causal factor could be an aspect of innate immunity or a genetically determined influence on the susceptibility to infection. **C**. The stronger the antibody response to the vaccine, the lower the infection risk (light blue trapezoid). This protective effect is counteracted by an infection-enhancing effect (dark blue), e.g. inflammation or greater proliferation of target cells upon viral exposure (cf. [22]). Since both effects are caused by the HIV-1 immunogens, the infection risk does not vary with the irrelevant Ab response in the placebo group. **D**. The Ab responses are indirect markers of a vaccine-induced protective effect that is not mediated by antibodies. As in A, a high HIV-1-specific Ab response is associated with a reduced risk of infection, a low response with an increased risk. As in B these Abs are not causally responsible. Unlike in B, however, they are markers of an immune factor that is induced by the vaccine. The infection risk, therefore, does not vary with the irrelevant Ab response among the placebo controls, but it does vary with both the irrelevant and the HIV-1-specific Ab responses among the vaccinees.

In the clinical trials Vax003 (a cohort of intravenous-drug abusers) and Vax004 (sexually exposed subjects), volunteers were immunized with recombinant outer envelope glycoprotein, gp120, from two HIV-1 strains. Although the vaccine did not protect in either trial (0-6% vaccine efficacy), post-trial analyses of serum samples from the Vax004 cohort showed that Ab reactivity with the CD4-binding site (CD4bs) on gp120, as well as antibody-dependent cell-mediated viral inhibition (ADCVI), correlated inversely with the risk of infection [3,21]. One explanation would be that weak anti-gp120 antibody (Ab) responses increased the rate of infection, while strong responses reduced it, the net infection rate approximating that for controls. Another explanation would be that Ab responses to gp120 were associated with an innate immune or other resistance variable that is not affected by the vaccine and that protects at high but not at low levels. Had this variable been known, it should have correlated with infection rates similarly in the vaccinee and placebo groups. Yet another possibility is that the vaccine, through a mechanism distributed over the entire group of vaccinees, increased susceptibility to infection (cf. [22]), but that such an effect was counteracted by the stronger anti-gp120 responses. Finally, the high or low reactivities with gp120, or both, could be mere markers of other vaccine-induced responses that directly reduced or increased the infection rate, respectively (Figure 1). 

The vaccine in the STEP trial consisted of an adenovirus vector, carrying the HIV-1 genes *gag, pol,* and *nef,* and was intended to induce cellular immunity. Vaccinees and controls who became infected got similar viral loads, but vaccination was associated with a modestly increased risk of infection [2,23]. Intriguingly, the increased infection risk correlated weakly with pre-existing Ab titers to the adenovirus vector among uncircumcised male vaccinees, while such titers inversely correlated with the risk of infection in the control group [24]. A partly analogous effect was later observed in a macaque experiment: monkeys that were chronically infected with a host-range mutant of adenovirus type 5 (Ad5) and then immunized with a replication-defective Ad5 SIVmac239 Gag-Pol-Nef vaccine showed increased susceptibility to a sub-preputial penile inoculation with low-dose SIVmac251. Unlike in the STEP trial, however, the acute-phase viral loads were reduced in the vaccinated macaques [18]. In another simian study, a trivalent Ad5 Gag-Pol-Nef vaccine neither protected against rectal challenge with repeated low-dose SIVsmE660 nor dampened viral loads, thereby also partly reproducing the STEP trial results [25]. The early post-vaccination CD8^+^ and CD4^+^ T-cell responses were broad but the anamnestic post-infection responses were narrow, perhaps an indicator of why there was no control of virus replication [25].

The vaccine regimen in the RV144 trial involved priming with a canarypox vector expressing the HIV-1 Gag, Pol and partial Env proteins (gp120 was directly fused to the transmembrane domain of gp41), and then boosting with gp120 derived from viruses of Clades B and E [5]. In contrast to its Vax and STEP predecessors, the RV144 trial did show some protection from infection (31%), but this was significant (p = 0.04) only when subjects who had become infected between enrollment and the first immunization were excluded from the vaccinee and placebo groups. Without that criterion, and also when only the participants who received all the intended immunizations are considered, efficacy was weaker and non-significant (26%, p = 0.08 and 0.16, respectively). Thus, the outcome of the trial was rather fragile. The apparent vaccine efficacy declined from 60% for the first year to 31% for the 3.5-year post-vaccination period, although with wide confidence intervals because of the small number of infections [5,26,27]. Since Ab titers to gp120 vaccines decline rapidly [3,15], it might be conjectured that the peak responses contribute to the higher efficacy at the earlier times. There is, however, an alternative explanation for the weakening efficacy of a vaccine [27]. Thus, if the exposure of the study participants to the virus or their susceptibility to infection varies equally within each group, high-risk subjects will be depleted earlier among placebo recipients than vaccinees, provided the vaccine has any activity against high-risk transmission events. The infection-rate ratio for vaccinees over placebo recipients will then increase, i.e. the apparent vaccine efficacy will decline over the follow-up period. Unlike the waning of a protective adaptive immune response, this predicted behavior of the cohort also explains why there was a declining infection rate in the RV144 placebo group [27]. The above epidemiological phenomenon, therefore, weakens any evidence for a causal link between changes in the immune response and apparent protection. It cannot invalidate the overall difference in infection rate between the vaccine and placebo groups, but it does encourage a search for innate-immune or other susceptibility factors that modulate infection rates in both groups. As indicated above, the intriguing associations observed in the results of three trials (Vax004, STEP, and RV144; the Vax003 results were not analyzed in a relevant manner) should motivate this kind of investigation.

### Augmenting the design of both trials and experiments

To improve the interpretation of vaccine-induced protection, Follmann has proposed two amplifications of trial design [28]. The first stratagem involves giving all participants an irrelevant vaccination at baseline and quantifying the responses to that immunogen. Alternatively, non-HIV-1 proteins expressed from a viral vector, given to both the vaccine and placebo groups, could serve as irrelevant immunogens in both humans and animals. Correlating the responses to the HIV-1 and irrelevant immunogens among the vaccinees would then allow a prediction of how the placebo subjects would have responded to the HIV-1 immunogen had they received it, i.e. the counterfactual scenario. At the end of the trial, the additional information would differentiate between two distinct hypotheses of how immune responses to the HIV-1 vaccine relate to protection. First, if the HIV-1-specific immune response were a CoP, the infection rate among the placebo subjects should not vary with the irrelevant response, and therefore not with the projected response they would have had to the HIV-1 immunogen. Second, if instead the vaccinees’ responses to the HIV-1 immunogen were merely an indirect marker for inherent resistance to infection (including innate responses not elicited by the HIV-1 immunogens), then the infection rates would be correlated with the irrelevant responses to similar extents in the two groups (Figure 1).

The irrelevant-immunogen approach would only work when the HIV-1-specific and irrelevant responses correlate so strongly that the former can be counterfactually projected from the latter in the placebo group (Figure 1). A second stratagem was therefore devised in which, at the end of the trial, all the uninfected placebo subjects would receive exactly the same HIV-1-vaccine regimen as the vaccinees got at the start [28]. Then, provided each subject’s responsiveness to the vaccine is constant over the time span of the trial, and if the HIV-1 immunogen elicited a CoP, the responses of the uninfected placebo recipients and the total vaccinee group should be similar, i.e. lower than for uninfected vaccinees and higher than for the subsequently infected vaccinees. And if the responses to the HIV-1 immunogen were merely markers of reduced susceptibility to infection, the uninfected subjects in the two groups should react similarly, i.e. both subsets should have stronger responses than the subsequently infected vaccinees had at the corresponding time after immunization.

The logistic complexities, and costs, of these augmented trial designs would surely be formidable. But their application might have differentiated between a causal and a susceptibility-marker hypothesis to explain the inverse association between infection risk and the magnitude of the anti-gp120 response in the Vax004 trial in which the vaccine was not protective overall [3,21]. In contrast, the surprising outcome of the STEP trial - that the responses to the adenovirus vector were associated with increased risk among vaccinees and decreased risk among the placebo recipients - illustrates a potential pitfall of the irrelevant-immunization approach. Thus, ostensibly irrelevant immunogens such as an adenovirus vector might actually affect susceptibility to infection differently in the trial groups. Regarding the RV144 trial, as for Vax004, questions remain about any causal role for Ab responses that correlate inversely with the risk of infection [17]. Specifically, however, if the weak Ab responses to the RV144 Env immunogens contributed to the slight net protection, they might have done so only in a subset of volunteers with other, particularly beneficial, adaptive or innate immune responses. Composite effects of this nature might have been discernible had the trial design been augmented.

### Searching for correlates of protection in case–control analyses of human trials

When the natural immune-mediated eradication of viral infection is followed by life-long immunity, as happens with many common viruses [15], vaccine development has a target to aim at: the immune correlates of protection. But this does not apply to HIV-1, for no instance of its immune clearance has been proven despite the many millions of infections. The closest analogues of natural immunity available are therefore the immune responses to chronic HIV-1 infection. Furthermore, the genetic divergence among HIV-1 strains makes superinfection readily detectable, which allows investigations of whether the responses to chronic infection protect. The results are daunting: superinfection rates are similar to primary infection rates in comparable populations [29,30]. Furthermore, at the time of infection, NAb titers are not consistently lower in individuals who acquire superinfection than those who do not [31]. Suppose, however, the strongest NAb responses of infected individuals really do reduce the risk of superinfection. The overall lack of protection against superinfection across a cohort might then be explained by the counteracting effect of a raised susceptibility to a second HIV-1 infection. Chronic inflammation and damaged innate immunity in already infected people are examples of how such an increase in susceptibility could arise.

Even against such a complex background, it is informative to compare the NAb responses of those who acquire superinfection with the neutralization capacity conferred by protective passive immunization, with the corresponding NAb responses to vaccines in macaque experiments, and with the humoral responses that correlate inversely with the risk of infection in vaccine trials and that have been implicated as protective.

Neutralizing titers of macaque plasma after passive immunization with polyclonal NAbs confer different degrees of protection against intravenous challenge with 75 TCID_50_ of SHIV-DH12. Thus, an EC_50_ in the TZM-bl assay of 1/4500 corresponded to 90% *in vivo* protection, while one of 1/230 gave 50% [32]. These neutralization titers may be compared with those against viruses superinfecting humans: in the same assay, the EC_50_ titers of autologous sera from soon before the superinfection event ranged from undetectable to 1/1000 and were similar for matched controls who did not become superinfected [31]. The lack of definite protection against human superinfection might, therefore, have been predicted by extrapolation from the macaque passive immunization experiments. The routes of infection in the two scenarios are, of course, different - the human subjects were heterosexually exposed women, whereas the macaques were challenged intravenously. Furthermore, the plasma titer of infused NAbs did not correlate with protection of macaques against vaginal challenge by 600 TCID_50_ of SHIV-89.6PD, but vaginal fluid NAb concentrations did correlate [20]. The NAb titers in the vaginal fluids of superinfected women are not known, although in general such titers are markedly lower than those in plasma [33]. Overall, we know too little about human superinfection to infer what local levels of vaccine-induced NAbs might minimally be required to prevent mucosal infection.

In the RV144 trial, IgG reactive with the hyper-variable gp120 V1V2 region was detected at slightly higher levels in plasma from uninfected vaccinees than from those who later became infected. This outcome occurred only with a scaffolded V1V2 fragment as the ELISA antigen, and not with V2 peptides or V1V2-containing gp120s. The association between V1V2 Abs and reduced risk was dampened by the presence of IgA Abs that bound to a soluble uncleaved gp140 protein, or to a gp120 C-terminal peptide. Nevertheless, this IgA reactivity was not itself associated with an increased risk of infection for vaccinees compared with placebo controls [17]. Also, among the lowest Env-IgA responders, both ADCC (Ab-dependent cellular cytotoxicity) and NAbs effective against sensitive HIV-1 strains were weakly associated with protection. It was therefore hypothesized that IgA molecules counteract IgG effector functions [17]. Such a contention is, however, problematic. Since the epitope specificities of the two isotypes differ, the molecular basis for any such blocking effect is unclear. Moreover, anti-Env IgA titers are considerably lower than the corresponding IgG titers, raising questions of how IgA could effectively block IgG binding. It should also be recalled that any Ab that can compete with a NAb for binding to functional Env trimers is likely itself to be neutralizing [34]. The hypothesis that V1V2-reactive IgG conferred protection also raises important questions: How high are these V1V2-binding capacities of IgG compared with those obtained in the Vax004 trial? There, Env binding was much stronger, but there was no protection against sexual transmission. How strong and cross-reactive are the RV144 V1V2 Ab responses compared with those elicited by infection, during which there is no net protection against superinfection?

It might be argued that because of how Env-reactive IgA Abs statistically modulated the infection risk in the RV144 trial, the CoP would be high V1V2-reactive IgG combined with low Env-reactive IgA [35]. But then again the question is whether Vax004 vaccinees and superinfected individuals did not have comparable or greater V1V2-IgG over Env-IgA ratios prior to the relevant infection events. If they did, why were they not protected?

How might anti-V1V2 antibodies protect? Or if they do not directly protect, are they associated with a mCoP? It should be recalled that if Ab binding to the V1V2 region of functional Env reaches a sufficient occupancy then HIV-1 infectivity is neutralized [34,36], and yet the NAb responses in RV144 sera were so weak, narrow, and infrequent as to lack protective potential [16,17]. One proposal is that V1V2 Abs block the binding of HIV-1 to the gut-homing integrin α4β7, the latter interaction hypothetically promoting cell-to-cell spread and the dissemination of newly transmitted founder viruses [17,37]. This idea has been undermined by recent observations that Abs to integrin α4β7 that block Env binding do not prevent founder viruses from replicating *in vitro*; only exceptional strains of little relevance to transmission are inhibited [38].

### Correlates of protection in the non-human primate model

Many experiments in non-human primate models have yielded extensive information on the immunogenicity and virological effects of various lentiviral vaccines [39-42]. Here, we highlight a small subset of studies that have provided robust evidence on correlates of risk. These experiments have included sufficient numbers of animals for definitive outcomes, and have used repeated low-dose viral challenges, thereby adding further statistical power. One such large-scale vaccine-efficacy study in macaques yielded some clues to CoRs and CoPs. The vaccination consisted of DNA priming with recombinant adenovirus boosting, both vectors expressing the SIVmac239 *gag-pol* and *env* genes [9]. The 129 Indian-origin rhesus monkeys were divided according to their expression of MHC Class I alleles associated with effective T-cell responses. Only monkeys lacking such alleles received the neutralization-resistant isolate, as a multiple low-dose rectal challenge. The vaccine did not protect significantly against acquisition but did suppress viral loads. In contrast, monkeys challenged with the more neutralization-sensitive SIVsmE660 strain were significantly protected against acquisition regardless of MHC genotype, but only those with favorable Class I alleles showed signs of viral-load suppression after infection.

Thus, the vaccine did not protect from infection with a challenge virus, SIVmac251, closely related to the vaccine strain, but did protect against a genetically more distant one, SIVsmE660. Both the immunological sensitivity and the replicative capacity of the two viruses could influence whether protection from infection occurs. A post-hoc analysis of another genetic host factor, *TRIM5* polymorphism, revealed that infection-restrictive genotypes were associated with protection against infection in both the vaccine and control groups [9]. Animals with the restrictive *TRIM5* genotypes were nearly all protected from acquisition by the vaccine, but those with permissive *TRIM5* genotypes also had significantly lower infection rates than the corresponding controls, which had a particularly high rate of infection.

The relative contributions to prevention of infection by several adaptive and innate immune variables were also evaluated by logistic regression. Among animals with favorable MHC alleles, the restrictive *TRIM5* genotypes were strongly associated with protection from infection, while for the other macaques NAbs and Env-specific CD4^+^ T-cells were the best correlates [9]. The dependency of vaccine efficacy on the genetic make-up of the animals suggests that protection mechanisms are multi-factorial.

A different vector-based SIV vaccine protected against infection by the robust SIVmac251 strain in a study where the frequencies of restrictive and permissive *TRIM5* genotypes were balanced among the groups [6]. Gag-Pol proteins, with or without Env, were expressed from different combinations of DNA, modified vaccinia Ankara (MVA) virus, and adenovirus vectors Ad26 and Ad35. Protection against SIVmac251 acquisition correlated with total anti-Env Ab signals, unlike protection against SIVsmE660 in the previous study, but was again linked to NAb responses, which were weak. A parallel with the human RV144 trial was suggested in that V2-specific Abs were associated with protection against infection, although the two studies used different V2-containing antigens and assays [6,17]. Moreover, anti-Env trimer titers also correlated with protection against infection [6]. It is unknown whether similar correlations would be observed for Abs to other SIV antigens (e.g., p24), as alternative measures of the humoral immune response to the vaccine. The magnitude and breadth of Gag CTL responses correlated with post-infection viremic control, but so did anti-Env Ab, tier-1 NAb, ADCC, ADCVI, and Env-specific CD4^+^ T-cell effector-memory responses. Of note is that ADCC, a potential protective mechanism for non-neutralizing Abs, was only weakly associated with protection against infection in the human RV144 trial but strongly linked to viral control in the macaque study [6,17]. Overall, important questions remain to be answered about which vaccine responses are protective mechanisms, which are only immune markers of susceptibility, and about how variables in either category are influenced by host genetics.

In a suggestive parallel to the Vax004 result, Env expressed from a replicating adenovirus-5 vector did not protect macaques against multiple low-dose SIVmac251 rectal challenges; but the infection rate in the Env-vaccine group was significantly lower for animals with high levels of Env-specific sIgA antibodies in rectal secretions than for those with low levels [43]. Rectal anti-Env sIgA antibodies were therefore suggested to be a protective mechanism [43]. As there was no overall protection in the experiment, however, the highest levels of rectal Env-specific sIgA antibodies could only have had any protective effect if the vaccine also exerted a counteracting effect that enhanced the rate of infection (Figure 1). The invocation of a counterbalance and a zero-sum effect are necessary, just as in the case of anti-gp120 responses in the non-protective Vax004 trial (see above).

In a study where macaques were immunized with DNA and a vaccinia vector expressing SIVmac239 Gag-Pol and Env, a correlation was found between the number of rectal SIVsmE660 challenges required for infection and an “avidity index” of Env-specific serum IgG [44]. The “avidity index” is commonly used [9,17,43]. It measures the capacity of Abs to bind Env in the presence of chaotropic ions and thus partly reflects Ab affinity for denatured Env proteins [45]. Ab reactivity with denatured Env could presumably only be an indirect marker of any mechanism that is active *in vivo*; if so, it would not qualify as an mCoP but could possibly be an nCoP. If, however, the imprecision of the “avidity index” and the very small range (30-45%) of its average values for animals infected after few or several challenges are taken into account [44], the predictive value of this variable vanishes. Furthermore, the subtle relationship between “avidity index” and infection status was only observed for animals that received the combined DNA-vaccinia immunizations: the macaques given only the vaccinia-based vaccine were equally well protected, but there was no correlation between risk and their “avidity index” values, which were higher than in the combination group [44]. It is of note that neither T-cell responses, rectal IgA, ADCC, nor NAbs correlated with protection in this study. In conclusion, the mCoP in this study remains unknown.

Since the RV144 case–control study has generated the hypothesis that V1V2-specific Abs are protective, it is worth analyzing vaccine regimens that have proven much more effective than RV144 while specifically excluding V2 epitopes from the immunogen. Complete protection of macaques from rectal SHIV-SF162P4 challenge was achieved through immunization with V2-deleted SF162 Env expressed from an alphavirus vector, followed by boosting with a soluble form of the V2-deleted Env trimer [46]. But the extent of the Ab responses associated with protection against infection overlapped considerably between protected and infected animals. Hence, for the most part, the Ab reactivities were not sufficient for protection. In a different study, an attenuated, V1V2-deleted variant of SIVmac239 conferred strong control of viremia when the macaques were challenged with the wild type virus [47]. Important anti-viral mechanisms clearly operate independently of V2 and probably target epitopes that are more conserved and hence more exploitable through vaccination.

### Cooperative immune functions

Active immunization engages multiple aspects of the immune system with innumerable inter-related effects. Synergistic mechanisms are difficult to identify in immune-correlates analyses; each component contributing to the synergy may be necessary but insufficient for the observed protection. And vaccine-induced responses might also synergize with, or be antagonized by, host variables that are unaffected by the vaccine.

After passive immunization, the prevention of SHIV infection by a broadly active NAb was enhanced by, but did not depend on, the Fc-receptor binding capacity of the IgG [48]. Protection might therefore sometimes depend on collaborations between NAbs and some cellular or other aspect of the immune system. Any damage to these other immune factors could help explain why pre-existing high levels of NAbs do not appear to protect already infected people against HIV-1 superinfection (see above).

Are vaccine effects on acquisition and viral loads completely independent? In animal experiments, ADCC has been associated with lower viral loads, just as have CTL responses [6]. Such similarities might be expected since both responses kill infected cells. Yet, in the RV144 trial there was a weak tendency for ADCC to be associated with protection against acquisition, whereas in the Vax004 trial ADCVI activity, like Env-binding Abs, was inversely associated with the risk of acquisition despite the lack of overall protection [21]. Both NAb and CTL responses were associated with lower viral loads after multiple rectal SHIV challenges of monkeys immunized with a combination of Env trimers and Gag-Pol particles [49]. In other studies, CTL responses did not enhance protection against infection by systemically infused NAbs [50], but combining an entry-inhibitor microbicide (which is comparable to mucosal NAbs) with a vaccine gave both lower viral loads and lower acquisition than either intervention alone [51]. When statistical associations are analyzed, each component in a multi-factorial, synergistic mCoP combination is likely to be imperfectly correlated with protection, because variation among the other factors will blur the relationships. The difficulty of identifying such combined causes may explain why mCoPs in human and monkey studies are so elusive.

## Conclusion

The search for inducible immune responses that protect against HIV-1 infection is delicate and error-prone, particularly when protection is weak or marginal [5,17]. If a vaccine only needed to elicit IgG recognizing with V1V2 epitopes with test-strain sequences, presented on monomeric scaffolds, the task of vaccination would be facile (and would surely have been accomplished already by gp120 vaccines). But it will be much harder if Abs cross-reacting with unknown V1V2 sequences - presented in their native trimeric form on the potentially transmitted virions - are required. Such antibodies would, *ipso facto*, be cross-neutralizing, which remains a prime goal of many vaccine strategies [52,53]. The assertion “RV144 vaccine data […] showed the protective ability of the V2 antibodies” [37] is completely unsubstantiated by the available data. The future may reveal that the hypothesis of protection by V1V2 antibodies is more of a cop-out than an outed CoP [54]. How and to what extent IgA of any specificity could counteract protective IgG responses is also utterly unclear. Overall, the RV144-inspired hypotheses of protection are hard to fit into an explanatory mechanistic framework. The possibility remains that indirect associations are being pursued, thereby distracting attention from the search for directly protective responses [54].

While acknowledging the logistic complications, we support Follmann’s arguments for augmenting the design of vaccine studies by administering irrelevant immunogens to both vaccinees and placebo subjects, and by performing post-trial vaccination among uninfected placebo recipients. These stratagems could be adopted in both human trials and animal experiments, for they would facilitate a sifting of causal correlates of protection from adventitiously associated factors. Some adaptive immune responses might only be effective when they act in unison with innate responses and constitutive resistance factors. Furthermore, if host factors that determine the strength of vaccine responses vary widely in a population, so will vaccine efficacy. The causes of such variation and of factors modulating susceptibility might be explored through systems-biological approaches, which have elucidated host determinants of effective vaccination against the influenza and yellow-fever viruses [55,56]. Genome-wide association studies of cohorts lacking protective *CCR5* variants have not yet identified host genotypes influencing HIV-1 acquisition, but complementary methods, such as large-scale sequencing, may prove fruitful [57,58]. Exploring the genetic basis for variable responses to HIV-1 immunogens may also be valuable. The host’s microbiome and previous exposures to immunogens might mold vaccine responses [59], in which case combinations of host-genome and microbiome traits could influence vaccine efficacy. In summary, a comprehensive causal analysis of protection will require a dissection of complex interactions among innate and adaptive immune responses as well as other host predispositions and environmental factors.

## Competing interests

The authors declare that they have no competing interests.

## Author contributions

PJK drafted the manuscript. PJK and JPM wrote the paper. Both authors read and approved the final manuscript.
